# Supplementary figures and images for: A DARPin-based molecular toolset to probe gephyrin and inhibitory synapse biology
Source: eLife. 2022 Oct 31;11:e80895. doi: 10.7554/eLife.80895 (PMC9674349; doi:10.7554/eLife.80895)

Full labelled Coomassie gel from Figure 1 H

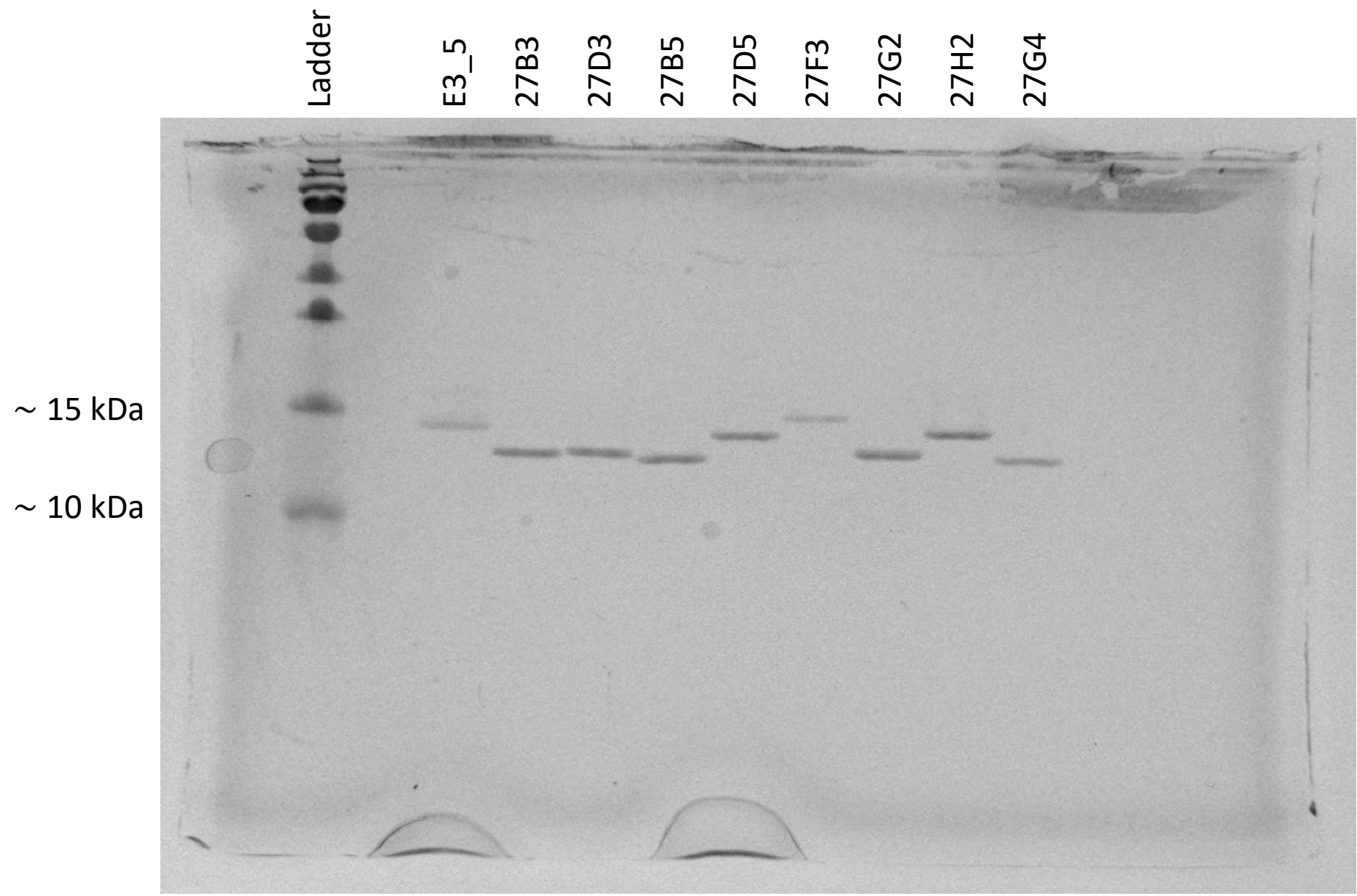

Supplement: Figure 1—source data 1. [file elife-80895-fig1-data1.pdf]

### DARPin-hFc structure

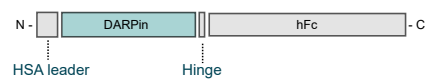

Supplement: Figure 3—figure supplement 5—source data 1. [file elife-80895-fig3-figsupp5-data1.pdf]

Immunoblot of gephyrin (detected using anti-mouse DyeLight 800)

~ 100 kDa

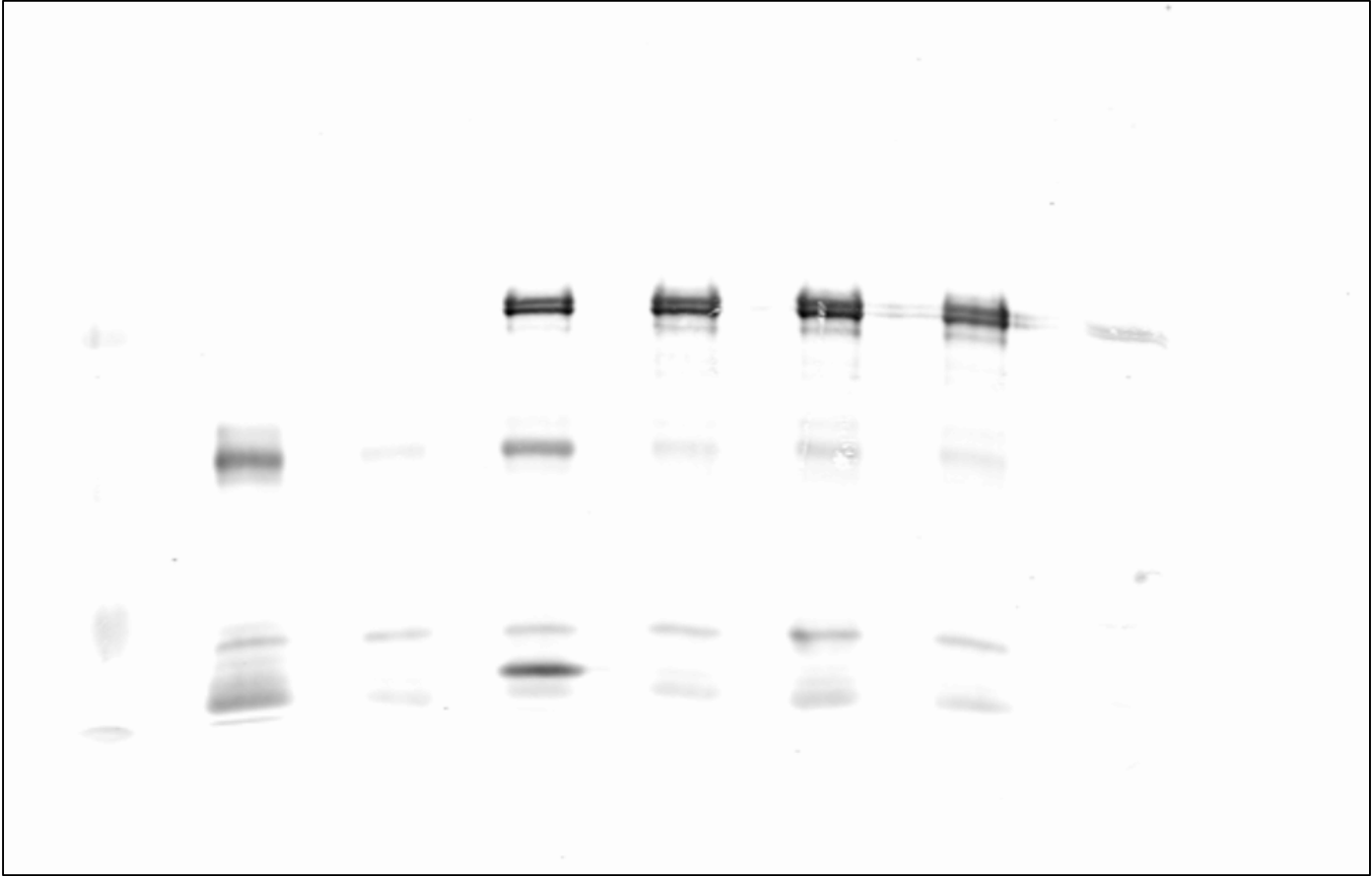

Supplement: Figure 5—figure supplement 1—source data 1. [file elife-80895-fig5-figsupp1-data1.pdf]
