# Supplementary material for: A DARPin-based molecular toolset to probe gephyrin and inhibitory synapse biology
Source: eLife. 2022 Oct 31;11:e80895. doi: 10.7554/eLife.80895 (PMC9674349; doi:10.7554/eLife.80895)
Supplement: Supplementary file 1. — Amino acid and nucleotide sequences are displayed for all characterized anti-gephyrin DARPins in this study. [file elife-80895-supp1.docx]

**Supplementary file 1: List of DARPin sequences.**

| Clone | | DNA Sequence | AA Sequence | DARPin ID | DARPin type |
| --- | --- | --- | --- | --- | --- |
| 27 | G2 | ATGAGAGGATCGCATCACCATCACCATCACCATCACGGATCCGACCTGGGTAAGAAACTGCTGGAAGCTGCTCGTGCTGGTCAGGACGACGAAGTTCGTATCCTGATGGCAAACGGTGCTGACGTTAACGCTATGGACTTCACTGGTTACACTCCGCTGCACCTGGCTGCTAAAGAAGGTCACCTGGAAATCGTTGAAGTTCTGCTGAAAACCGGTGCTGACGTTAACGCTATCGACAAACGTGGTAACACTCCGCTGCACCTGGCTGCTTGGCGTGGTCACCTGGAAATCGTTGAAGTTCTGCTGAAGCACGGCGCCGACGTTAACGCTCAGGACGTTTACGGTACTACTCCGTTCGACCTGGCTGCTTGGGCTGGTAACGAGGACATCGCTGAAGTTCTGCAGAAAGCTGCTAAGCTTAATGACTACAAGGATGACGACGACAAG | MRGSHHHHHHHHGSDLGKKLLEAARAGQDDEVRILMANGADVNAMDFTGYTPLHLAAKEGHLEIVEVLLKTGADVNAIDKRGNTPLHLAAWRGHLEIVEVLLKHGADVNAQDVYGTTPFDLAAWAGNEDIAEVLQKAAKLNDYKDDDDK | 008-855-2308-A9 | N2C |
| 27 | H2 | ATGAGAGGATCGCATCACCATCACCATCACCATCACGGATCCGACCTGGGTAAGAAACTGCTGGAAGCTGCTCGTGCCGGTCAGGACGACGAAGTTCGTATCCTGATGGCAAACGGTGCTGACGTTAACGCTTGGGACAAACATGGTCATACTCCGCTGCACCTGGCTGCTGCTTGGGGTCACCTGGAAATCGTTGAAGTTCTGTTGAAAACCGGTGCTGACGTTAACGCTCAGGACCAGATGGGTTACACTCCGCTGCACCTGGCTGCTTGGTACGGTCATCTGGAAATCGTTGAAGTTCTGCTGAAGCATGGCGCCGACGTTAACGCTCAGGACAAATTCGGTAAGACTCCGTTCGACCTGGCTGCTATGGCTGGTAACGAGGACATCGCTGAAGTTCTGCAGAAAGCTGCTAAGCTTAATGACTACAAGGATGACGACGACAAG | MRGSHHHHHHHHGSDLGKKLLEAARAGQDDEVRILMANGADVNAWDKHGHTPLHLAAAWGHLEIVEVLLKTGADVNAQDQMGYTPLHLAAWYGHLEIVEVLLKHGADVNAQDKFGKTPFDLAAMAGNEDIAEVLQKAAKLNDYKDDDDK | 008-855-2308-B9 | N2C |
| 27 | B3 | ATGAGAGGATCGCATCACCATCACCATCACCATCACGGATCCGACCTGGGTAAGAAACTGCTGGAAGCTGCTATCCATGGTCAGCTGGACGAAGTTCGTATCCTGATGGCAAACGGTGCTGACGTTAACGCTACTGACCTGCAGGGTCATACTCCGCTGCACCTGGCTGCTAAATGGGGTCACCTGGAAATCGTTGAAGTTCTGCTGAAAACCGGTGCTGACGTTAACGCTGAAGACGTTCGTGGTTACACTCCGCTGCACCTGGCTGCTCTGTGGGGTCACCTGGAAATCGTTGAAGTTCTGCTGAAGCACGGCGCCGACGTTAACGCTCAGGACCGTTGGGGTGAAACTCCGTTCGACCTGGCTGCTTGGTTCGGTAACGAGGACATCGCTGAAGTTCTGCAGAAAGCTGCTAAGCTTAATGACTACAAGGATGACGACGACAAG | MRGSHHHHHHHHGSDLGKKLLEAAIHGQLDEVRILMANGADVNATDLQGHTPLHLAAKWGHLEIVEVLLKTGADVNAEDVRGYTPLHLAALWGHLEIVEVLLKHGADVNAQDRWGETPFDLAAWFGNEDIAEVLQKAAKLNDYKDDDDK | 008-855-2308-C11 | N2C |
| 27 | D3 | ATGAGAGGATCGCATCACCATCACCATCACCATCACGGATCCGACCTGGGTAAGAAACTGCTGGAAGCTGCTCGTGCTGGTCAGGACGACGAAGTTCGTATCCTGATGGCAAACGGTGCTGACGTTAACGCTGTTGACACTTTCGGTTGGACTCCGCTGCACCTGGCTGCTGCTAACGGTCACCTGGAAATCGTTGAAGTTCTGCTGAAAACCGGTGCTGACGTTAACGCTAACGACCAGCGTGGTAACACTCCGCTGCACCTGGCTGCTTGGGACGGTCACCTGGAAATCGTTGAAGTTCTGCTGAAGCACGGCGCCGACGTTAACGCTCAGGACAACTTCGGTATCACTCCGTTCGACCTGGCTGCTTACCGTGGTAACGAGGACATCGCTGAAGTTCTGCAGAAAGCTGCTAAGCTTAATGACTACAAGGATGACGACGACAAG | MRGSHHHHHHHHGSDLGKKLLEAARAGQDDEVRILMANGADVNAVDTFGWTPLHLAAANGHLEIVEVLLKTGADVNANDQRGNTPLHLAAWDGHLEIVEVLLKHGADVNAQDNFGITPFDLAAYRGNEDIAEVLQKAAKLNDYKDDDDK | 008-855-2309-A6 | N2C |
| 27 | F3 | ATGAGAGGATCGCATCACCATCACCATCACCATCACGGATCCGACCTGGGTAAGAAACTGCTGGAAGCTGCTCGTGCTGGTCAGGACGACGAAGTTCGTATCCTGATGGCAAACGGTGCTGACGTTAACGCTTCTGACGTTGTTGGTCAGACTCCGCTGCACCTGGCTGCTTGGTCTGGTCACCTGGAAATCGTTGAAGTTCTGCTGAAAACCGGTGCTGACGTTAACGCTGAAGACATGGTTGGTAACACTCCGCTGCACCTGGCTGCTTACGTTGGTCACCTGGAAATCGTTGAAGTTCTGCTGAAGGCCGGCGCTGACGTTAACGCTGACGACTTCCGTGGTCGTACTCCGCTGCACCTGGCTGCTTACTACGGTCACCTGGAAATTGTTGAAGTTCTGCTGAAGCACGGCGCCGACGTTAACGCTCAGGACAAATTCGGTAAGACTCCGTTCGACCTGGCTATCGACAACGGTAACGAGGACATCGCTGAAGTTCTGCAGAAAGCTGCTAAGCTTAATGACTACAAGGATGACGACGACAAG | MRGSHHHHHHHHGSDLGKKLLEAARAGQDDEVRILMANGADVNASDVVGQTPLHLAAWSGHLEIVEVLLKTGADVNAEDMVGNTPLHLAAYVGHLEIVEVLLKAGADVNADDFRGRTPLHLAAYYGHLEIVEVLLKHGADVNAQDKFGKTPFDLAIDNGNEDIAEVLQKAAKLNDYKDDDDK | 008-855-2309-D7 | N3C |
| 27 | G4 | ATGAGAGGATCGCATCACCATCACCATCACCATCACGGATCCGACCTGGGTAAGAAACTGCTGGAAGCTGCTCGTGCTGGTCAGGACGACGAAGTTCGTATCCTGATGGCAAACGGTGCTGACGTTAACGCTGAAGACGCTAAAGGTCATACTCCGCTACACCTGGCTGCTTACCTGGGTCACCTGGAAATCGTTGAAGTTCTGCTGAAAACCGGTGCTGACGTTAACGCTTACGACAAACATGGTCATACTCCGCTGCACCTGGCTGCTTCTTGGGGTCACCTGGAAATCGTTGAAGTTCTGCTGAAGGCCGGCGCTGACGTTAACGCTTCTGACCATACTGGTCGTACTCCGCTGCACCTGGCTGCTTGGTACGGTCACCTGGAAATCGTTGAAGTTCTGCTGAAGGCCGGCGCTGACGTTAACGCTCAGGACAAATTCGGTAAGACTCCGTTCGACCTGGCTATCGACAACGGTAACGAGGACATCGCTGAAGTTCTGCAGAAAGCTGCTAAGCTTAATGACTACAAGGATGACGACGACAAG | MRGSHHHHHHHHGSDLGKKLLEAARAGQDDEVRILMANGADVNAEDAKGHTPLHLAAYLGHLEIVEVLLKTGADVNAYDKHGHTPLHLAASWGHLEIVEVLLKAGADVNASDHTGRTPLHLAAWYGHLEIVEVLLKAGADVNAQDKFGKTPFDLAIDNGNEDIAEVLQKAAKLNDYKDDDDK | 008-855-2310-G12 | N3C |
| 27 | B5 | ATGAGAGGATCGCATCACCATCACCATCACCATCACGGATCCGACCTGGGTAAGAAACTGCTGGAAGCTGCTTTCATGGGTCAGCACGACGAAGTTCGTATCCTGATGGCAAACGGTGCTGACGTTAACGCTCAGGACAAAGCTGGTCATACTCCGCTGCACCTGGCTGCTCAGATGGGTCACCTGGAAATCGTTGAAGTTCTGCTGAAAACCGGTGCTGACGTTAACGCTTCTGACTGGTACGGTATCACTCCGCTGCACCTGGCTGCTTGGAACGGTCACCTGGAAATCGTTGAAGTTCTGCTGAAGCACGGCGCCGACGTTAACGCTCAGGACTGGGACGGTAACACTCCGTTCGACCTGGCTGCTATGGTTGGTAACGAGGACATCGCTGAAGTTCTGCAGAAAGCTGCTAAGCTTAATGACTACAAGGATGACGACGACAAG | MRGSHHHHHHHHGSDLGKKLLEAAFMGQHDEVRILMANGADVNAQDKAGHTPLHLAAQMGHLEIVEVLLKTGADVNASDWYGITPLHLAAWNGHLEIVEVLLKHGADVNAQDWDGNTPFDLAAMVGNEDIAEVLQKAAKLNDYKDDDDK | 008-855-2311-B9 | N2C |
| 27 | D5 | ATGAGAGGATCGCATCACCATCACCATCACCATCACGGATCCGACCTGGGTAAGAACCTGCTGGAAGCTGCTGTTCAGGGTCAGGACGACGAAGTTCGTATCCTGATGGCAAACGGTGCTGACGTTAACGCTGAAGACTTCCATGGTCTGACTCCGCTGCACCTGGCTGCTTGGCATGGTCACCTGGAAATCGTTGAAGTTCTGCTGAAAACCGGTGCTGACGTTAACGCTCATGACATGATCGGTTGGACTCCGCTGCACCTGGCTGCTCGTGTTGGTCACCTGGAAATCGTTGAAGTTCTGCTGAAGGCCGGCGCTGACGTTAACGCTTGGGACACTCGTGGTCGTACTCCGCTGCACCTGGCTGCTTGGGCTGGTCACCTGGAAATCGTTGAAGTTCTGCTGAAGCACGGCGCCGACGTTAACGCTCAGGACAAATTCGGTAAGACTCCGTTCGACCTGGCTATCGACAACGGTAACGAGGACATCGCTGAAGTTCTGCAGAAAGCTGCTAAGCTTAATGACTACAAGGATGACGACGACAAG | MRGSHHHHHHHHGSDLGKNLLEAAVQGQDDEVRILMANGADVNAEDFHGLTPLHLAAWHGHLEIVEVLLKTGADVNAHDMIGWTPLHLAARVGHLEIVEVLLKAGADVNAWDTRGRTPLHLAAWAGHLEIVEVLLKHGADVNAQDKFGKTPFDLAIDNGNEDIAEVLQKAAKLNDYKDDDDK | 008-855-2311-D10 | N3C |
|  | E3_5 | ATGAGAGGATCGCATCACCATCACCATCACCATCACGGATCCGACCTGGGTAAGAAACTGCTGGAAGCTGCTCGTGCTGGTCAGGACGACGAAGTTCGTATCCTGATGGCTAACGGTGCTGACGTTAACGCTACTGACAATGATGGTTATACTCCGCTGCACCTGGCTGCTTCTAATGGTCACCTGGAAATCGTTGAAGTTCTGCTGAAGAACGGTGCTGACGTTAACGCTTCTGACCTTACTGGTATTACTCCGCTGCACCTGGCTGCTGCTACTGGTCACCTGGAAATCGTTGAAGTTCTGCTGAAGCACGGTGCTGACGTTAACGCTTATGACAATGATGGTCATACTCCGCTGCACCTGGCTGCTAAGTATGGTCACCTGGAAATCGTTGAAGTTCTGCTGAAGCACGGTGCTGACGTTAACGCTCAGGACAAATTCGGTAAGACCGCTTTCGACATCTCCATCGACAACGGTAACGAGGACCTGGCTGAAATCCTGCAAAAGCTTAATGACTACAAGGATGACGACGACAAG | MRGSHHHHHHHHGSDLGKKLLEAARAGQDDEVRILMANGADVNATDNDGYTPLHLAASNGHLEIVEVLLKNGADVNASDLTGITPLHLAAATGHLEIVEVLLKHGADVNAYDNDGHTPLHLAAKYGHLEIVEVLLKHGADVNAQDKFGKTAFDISIDNGNEDLAEILQKLNDYKDDDDK | E3_5 | N3C |
